# The use of memantine for depressive symptomatology

**DOI:** 10.1192/j.eurpsy.2024.1104

**Published:** 2024-08-27

**Authors:** J. T. Coelho, S. Timóteo, A. S. Machado

**Affiliations:** Department of Psychiatry and Mental Health, University Hospital Center of São João, Porto, Portugal

## Abstract

**Introduction:**

Depression is one of the most prevalent and incapacitating disease in current times and depressive symptoms have important global functioning implications.

The serotonergic and glutamatergic systems are involved in the pathophysiology and treatment of depression. Ketamine is an N-methyl-d-aspartic acid (NMDA) receptor antagonist that has demonstrated an important role on depressive symptoms, but its use is restricted due to its dissociative effects and other possible adverse effects.

Memantine is a noncompetitive antagonist of the NMDA receptor that modulates glutamate transmission. Memantine is used for the treatment of moderate to severe Alzheimer’s disease.

**Objectives:**

In this review, we aim to investigate, organize and synthetize the current data about the use of memantine for depressive symptoms.

**Methods:**

Our literature research focused on some of the most significative articles published in the last decade, including meta-analysis and systematic reviews.

**Results:**

Most of the relevant literature suggests that memantine may effectively reduce depressive symptoms in patients with mood disorders.

The literature also supports that memantine’s glutamatergic mechanism of action could reduce apathy and treat depression comorbid with alcohol abuse.

Memantine affects brain-derived neurotrophic factor(BDNF) production suggesting that glutamate assumes an essential role in the pathology and etiology of depression. Also, the relationship between depression and the NMDA receptor is further supported by the fact that people with major depressive disorder demonstrate higher glutamate levels in the brain and blood.

Moreover, current studies demonstrate that treatment with memantine as adjunct to selective serotonin reuptake inhibitors (namely sertraline) manifested a favourable safety and efficacy profile in patients with major depressive disorder.

**Conclusions:**

Memantine may have a wide therapeutic use beyond its utility in neurodegenerative diseases.

More studies should be performed, especially larger controlled studies of longer duration focusing on long-term safety and efficacy.

**Disclosure of Interest:**

None Declared

